# *Lactobacillus acidophilus* KBL409 Ameliorates Atopic Dermatitis in a Mouse Model

**DOI:** 10.1007/s12275-024-00104-5

**Published:** 2024-02-22

**Authors:** Woon-ki Kim, You Jin Jang, SungJun Park, Sung-gyu Min, Heeun Kwon, Min Jung Jo, GwangPyo Ko

**Affiliations:** 1 Department of Environmental Health Sciences, Graduate School of Public Health, Seoul National University, Seoul, 08826 Republic of Korea; 2 Institute of Health and Environment, Seoul National University, Seoul, 08826 Republic of Korea; 3 N-Bio, Seoul National University, Seoul, 08826 Republic of Korea; 4 KoBioLabs, Inc, Seoul, 08826 Republic of Korea

**Keywords:** Atopic dermatitis, Gut-skin axis, Immune response, *Lactobacillus acidophilus*, Microbiota, Probiotic

## Abstract

Atopic dermatitis (AD) is a chronic inflammatory skin disease with repeated exacerbations of eczema and pruritus. Probiotics can prevent or treat AD appropriately via modulation of immune responses and gut microbiota. In this study, we evaluated effects of *Lactobacillus acidophilus* (*L. acidophilus*) KBL409 using a house dust mite (*Dermatophagoides farinae*)-induced in vivo AD model. Oral administration of *L. acidophilus* KBL409 significantly reduced dermatitis scores and decreased infiltration of immune cells in skin tissues. *L. acidophilus* KBL409 reduced in serum immunoglobulin E and mRNA levels of T helper (Th)1 (Interferon-γ), Th2 (Interleukin [IL]-4, IL-5, IL-13, and IL-31), and Th17 (IL-17A) cytokines in skin tissues. The anti-inflammatory cytokine IL-10 was increased and Foxp3 expression was up-regulated in AD-induced mice with *L. acidophilus* KBL409. Furthermore, *L. acidophilus* KBL409 significantly modulated gut microbiota and concentrations of short-chain fatty acids and amino acids, which could explain its effects on AD. Our results suggest that *L. acidophilus* KBL409 is the potential probiotic for AD treatment by modulating of immune responses and gut microbiota of host.

**Supplementary Information:**

The online version contains supplementary material available at 10.1007/s12275-024-00104-5.

## Introduction

Atopic dermatitis (AD) is a common chronic inflammatory skin disease with repeated exacerbations of eczema and pruritus. In recent years, the prevalence of AD is steadily increasing (Petersen et al., 2018; Won et al., 2011). Recent studies have been reported that immunologic aberrations and gut microbiota alterations can contribute to the onset and the development of AD (Marsella & De Benedetto, 2017; Petersen et al., 2018). Skin tissues of patients with AD have high concentrations of immunoglobulin E (IgE) and display infiltration of lymphocytes, mast cells, eosinophils, and neutrophils (Fania et al., 2022; Marsella & De Benedetto, 2017). In the acute phase of AD, inflammatory responses are mediated by T helper (Th) 2 cells, which modulate the IgE concentration (Fania et al., 2022). In the chronic phase of AD, inflammatory responses from Th1, Th2, and Th17 cells are enhanced (Fania et al., 2022). Th2 cells induce inflammatory cytokines, including IL-4, IL-5, IL-13, and IL-31, which can affect allergic responses in skin (Fania et al., 2022; Kim et al., 2019a, 2020; Szegedi et al., 2012). IL-4 and IL-13 promote IgE class switching and B-cell production (Fang et al., 2021; Lee et al., 2016). IL-5 recruits and activates eosinophils (Won et al., 2011). IL-31 can disrupt the skin barrier and cause itching (Szegedi et al., 2012).

Live biotherapeutic products (LBPs) are live microorganisms with intended therapeutic or preventive effects. LBPs can ameliorate various diseases by modulating the immune responses (Round & Mazmanian, 2009; van Baarlen et al., 2013). Moreover, LBPs have beneficial health effects for host through modulation of gut microbiota (Han et al., 2022; Jang et al., 2019; Kim et al., 2019b; Sánchez et al., 2017). Oral administration of *Lactobacillus* spp., which is a well-known probiotic species, can improve symptoms of AD (Fang et al., 2021; Kim et al., 2019a, 2020). Moreover,  *Lactobacillus acidophilus* (*L. acidophilus*) KBL409, which was isolated from healthy Korean feces, reduced immune responses, restored gut microbiota and produced beneficial metabolites in mouse models of colitis and kidney fibrosis (Kim et al., 2021, 2022b).

Recently, the ‘gut-skin axis’ concept, which is indicating that a close relationship between host gut microbiota and skin conditions, has been implied as the potential target for AD treatment (Fang et al., 2021). Patients with AD showed reduction of gut microbial diversities and increase in harmful bacteria compared to healthy individuals (Fang et al., 2021). Probiotics can ameliorate the severity of AD symptoms by restoring intestinal dysbiosis and replenishing important metabolites, such as amino acids (AAs) and short-chain fatty acids (SCFAs) (Fang et al., 2021; Petersen et al., 2018). Especially, SCFAs, including acetate, propionate, and butyrate, are mainly produced during the fermentation process of gut microbiota using dietary fiber of resistant starch (Portincasa et al., 2022). SCFAs exert immunomodulatory effects and enhance the gut epithelial barrier to maintain gut homeostasis (Portincasa et al., 2022).

In this study, we investigated effects of *L. acidophilus* KBL409 in an in vivo AD-induced mouse model. We evaluated improvements of AD symptoms, changes in Th1, Th2, and Th17 cytokines and gut microbiota, and productions of metabolites due to oral administration of *L. acidophilus* KBL409 to suggest the potential probiotic for AD treatment.

## Materials and Methods

### Cultivation of *L. acidophilus* KBL409

*L. acidophilus* KBL409, isolated from the feces of healthy Koreans, was anaerobically cultured at 37 °C for 24 h using *Lactobacilli* MRS broth (BD Difco) with 0.05% L-cysteine hydrochloride (Kim et al., 2021). *L. acidophilus* KBL409 was identified using 16S ribosomal RNA gene sequencing and showed strong resistances in high concentrations of bile salts and low pH conditions (Kim et al., 2021). Prior to use, bacterial cells were collected by centrifugation (1,200 ×*g*) for 10 min and washed twice with 1× phosphate-buffered saline (PBS). Bacterial concentration was measured using a cultivation method and suggested as colony forming units (CFU)/ml.

### House dust mite (*Dermatophagoides farinae*, DFE)-Induced AD Mouse Model

A DFE-induced AD mouse model was performed as described in previous studies (Kim et al., 2019a, 2020; Meng et al., 2019; Sawada et al., 2007) with some modifications. Briefly, 5-week-old male NC/Nga mice (Central Lab Animals Inc.) were prepared and randomly assigned to experimental groups (n = 9 per group). Three mice were placed in an air-conditioned cage under a 12 h/12 h light/dark cycle. All mice can access water and food ad libitum. Dorsal and ear skin hair of mice were removed and 150 µl of 4% sodium dodecyl sulfate (SDS) was applied for 3 h to disrupt skin barrier. Then, 100 mg of *Dermatophagoides farinae* extract (DFE) cream (Biostir, Inc) was applied twice a week for 21 days to induce AD. Subsequently, approximately 1 × 10^9^ CFU of *L. acidophilus* KBL409 in 200 µl of 1× PBS was administered to mice daily by oral gavage. During administration, 100 mg of DFE cream was applied on once a week to maintain AD induction and prevent severe AD symptoms, which can occur the sudden deaths of mice. After 28 days of *L. acidophilus* KBL409 administration, all mice were euthanized. Blood, cecum, and skin samples were collected for further analyses.

### Evaluation of AD Symptoms

We evaluated changes of skin thickness due to *L. acidophilus* KBL409 administration using images of the ear or dorsal skin of each mouse. Moreover, we measured dermatitis scores once a week with following criteria: (1) erythema/hemorrhage, (2) scaling/dryness, (3) edema, and (4) excoriation/erosion as previously described (Table S1) (Hanifin et al., 2001; Kang et al., 2015; Kim et al., 2019a). Dermatitis scores were calculated using the average of scores from four researchers.

To measure immunoglobulin E (IgE) concentration, serum was separated from blood using centrifugation (1,200 ×*g*, 15 min, 4 °C). An IgE enzyme-linked immunosorbent assay (ELISA) kit (Koma Biotech) was used to confirm serum IgE concentrations according to the manufacturer’s instructions.

### Histological Analysis

Dorsal skin tissues (thickness: ~ 5 μm) were fixed in 10% neutral buffered formalin for 24 h and stained hematoxylin and eosin (H&E staining), as described previously (Kang et al., 2015; Kim et al., 2019a). A Panoramic Viewer (3DHISTECH, Ltd.) were used to examine stained tissues (Kim et al., 2019a, 2020).

### Measurement of mRNA Levels of Cytokines and Foxp3 in Skin Tissues

mRNA levels for various cytokines and Foxp3 were measured as described previously (Kim et al., 2019a). Briefly, total RNA of skin tissues was extracted using an Easy-spin Total RNA Extraction Kit (iNtRON Biotechnology) and complementary DNA was synthesized using a High-Capacity RNA to-cDNA Kit (Thermo Fisher Scientific) according to the manufacturer’s instructions. Then, quantitative PCR reactions were performed using a Rotor-Gene Q (Qiagen) with a QuantiTect SYBR Green PCR kit (Qiagen) and 0.01 mM primers (total volume: 20 μl; Table S2) under following conditions: initial denaturation at 95 °C for 10 min; followed by 40 cycles of 95 °C for 5 s and 60 °C for 10 s, as described previously with minor modification (Kim et al., 2019a). Relative expressions of target genes were calculated using the 2^−ΔΔCt^ method and normalized to the level of hypoxanthine–guanine phosphoribosyltransferase (HPRT) (Livak & Schmittgen, 2001).

### Analysis of Cecal Microbiota

Microbiota in cecum samples were analyzed as described previously with minor modifications (Kim et al., 2019a). Briefly, total genomic DNA was extracted using a QIAamp Fast DNA Stool Mini Kit (Qiagen) according to the manufacturer’s instructions. The V4–V5 hypervariable regions of 16S rRNA genes were amplified using the universal primers 515F and 926R. Amplicons were purified using a QIAquick PCR Purification Kit (Qiagen). 16S rRNA gene sequencing was performed using a MiSeq platform (Illumina Inc.).

Data were analyzed using Quantitative Insights into Microbial Ecology (QIIME)2 software (ver. 2022.2; QIIME 2 development team) with Greengenes ver. 13_8 database, as described previously (Han et al., 2022). Sequences were initially clustered into operational taxonomic units (OTUs) with at least 97% of nucleotide identity. Singletons and rare OTUs were excluded and relative abundances of microbial taxa were achieved using a table of non-rarefied OTUs. Alpha and beta diversities were suggested using Shannon index and Bray–Curtis distance-based principal coordinates analysis, respectively. Linear discriminant analysis effect size (LEfSe) analysis was performed using Galaxy ver. 2.0 (Hutlab). Phylogenetic investigation of communities by reconstruction of unobserved states (PICRUSt)2 ver. 2.4.1 analysis was performed using the Kyoto Encyclopedia of Genes and Genomes (KEGG) orthologous gene family database (Kanehisa Laboratories, Kyoto University) (Langille et al., 2013).

### Measurement of Metabolite Concentrations in Cecum Samples

SCFAs in cecum samples were analyzed as described previously (Kim et al., 2019a, 2020). First, homogenized cecum samples in distilled water were centrifuged at 13,000 ×*g* for 5 min. Supernatant was collected and internal standards (1% 2-methylpentanoic acid for volatile acids or benzoic acid for non-volatile acids, respectively) were applied. Subsequently, extraction solvents (ethyl ether for volatile acids or chloroform for non-volatile acids, respectively) were applied and resulting solution was centrifuged at 13,000 ×*g* for 5 min. The organic layer was analyzed using an Agilent 7890A gas chromatograph (Agilent Technologies) under following conditions; 1.5 kV of capillary voltage, 600 L/h of desolvation gas flow, 50 L/h of cone gas flow, 170 °C of oven temperature, and 225 °C for a flame ionization detector and an injection port temperature. A standard mixture was used to identify SCFAs (David et al., 2014).

AAs in cecum samples were measured as described previously (Kim et al., 2019a, 2020). Briefly, 1 ml of cecum extract were prepared in liquid chromatography-grade methanol (20 mg/ml). Then, 70 μl of AccQ•Tag Ultra Borate Buffer (Waters Corporation) and 20 μl of AccQ•Tag Ultra reagent (Waters Corporation) were subjected to derivatization for 10 min at 55 °C. An Acquity ultra-performance liquid chromatography (UPLC) (Waters Corporation) and a SYNAPT G2-Si mass spectrometer (Waters Corporation) with an ESI probe and MassLynx software 4.1 (Waters Corporation) were used under following conditions: 1.5 kV of capillary voltage, 600 L/h of desolvation gas flow, 50 L/h of cone gas flow, and 250 °C of desolvation temperature (Roucher et al., 2013). An AA-S-18 analytical standard mixture (Sigma-Aldrich.) were used to identify AAs.

### Statistical Analysis

All experimental data were expressed as the means ± standard deviation (SD) of at least three independent experiments. The Mann–Whitney *U* test was used to assess statistically significance (*P* < 0.05). GraphPad Prism ver. 7.00 (GraphPad Software, Inc.) was used for statistical analysis and visualization.

## Results

### Effects of *L. acidophilus* KBL409 on AD Symptoms

**Fig. 1:**
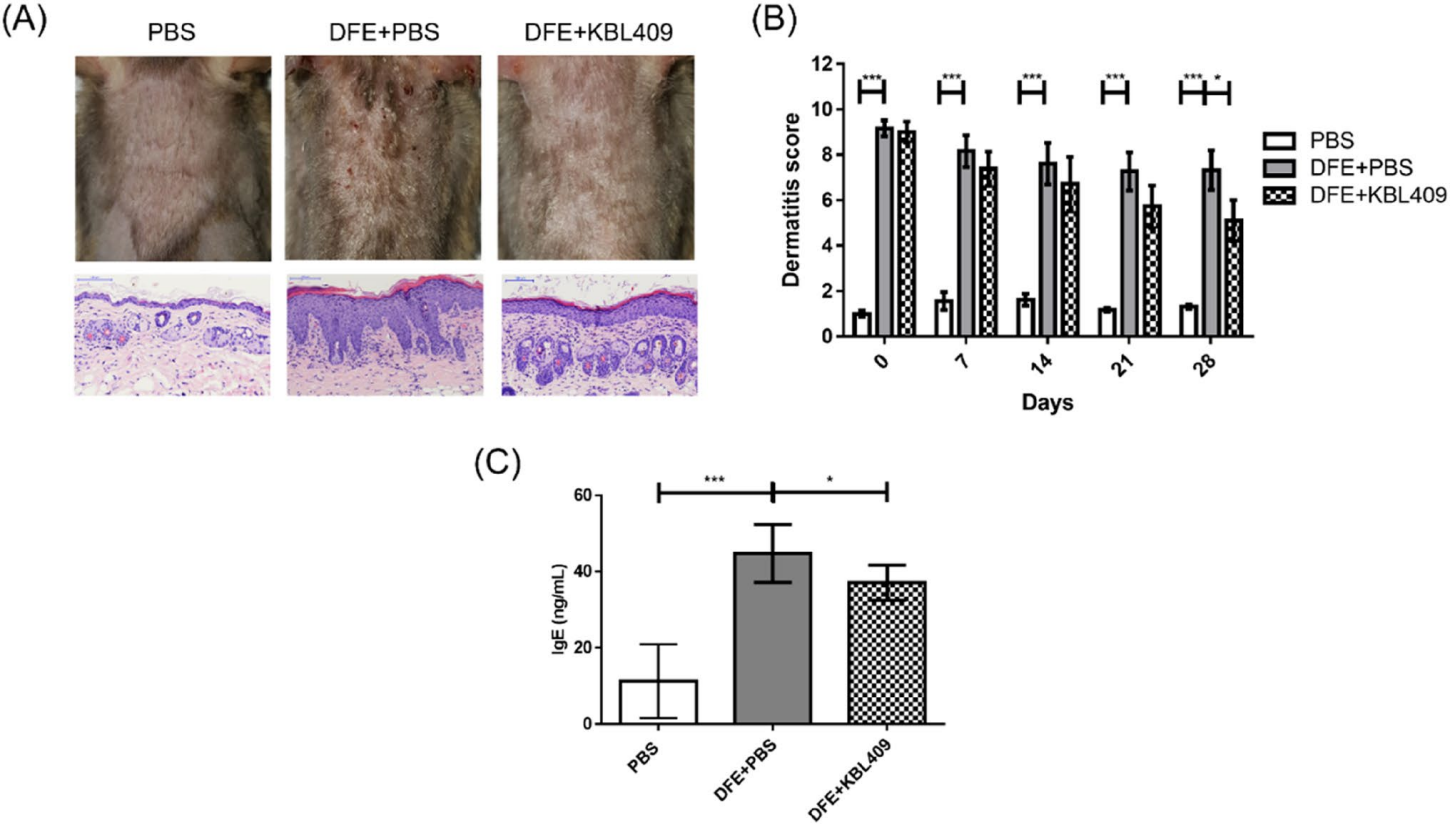
Effects of *L. acidophilus* KBL409 on AD symptoms. **A** Skin samples stained with hematoxylin and eosin; **B** Dermatitis score; **C** Serum IgE concentration. When appropriate, data are suggested as the mean ± standard deviation (SD) of experimental groups (nine mice per each group). Asterisks indicate a statistically significance (*P < 0.05; ***P < 0.001; the Mann–Whitney *U* test compared to the DFE + PBS-treated group)

After 28 days of administration, hyperkeratosis and epidermal thickness in ear or dorsal skin were reduced in DFE + *L. acidophilus* KBL409-treated mice (Fig. 1A). DFE + *L. acidophilus* KBL409-treated mice exhibited significantly lower dermatitis scores compared to the DFE + PBS treated group (5.11 ± 2.71; *P* < 0.05) (Fig. 1B). Serum IgE levels were significantly reduced after *L. acidophilus* KBL409 administration (37.01 ± 4.59 ng/ml; *P* < 0.05) (Fig. 1C).

### Effects of *L. acidophilus* KBL409 on mRNA Levels of Cytokines and Foxp3

**Fig. 2:**
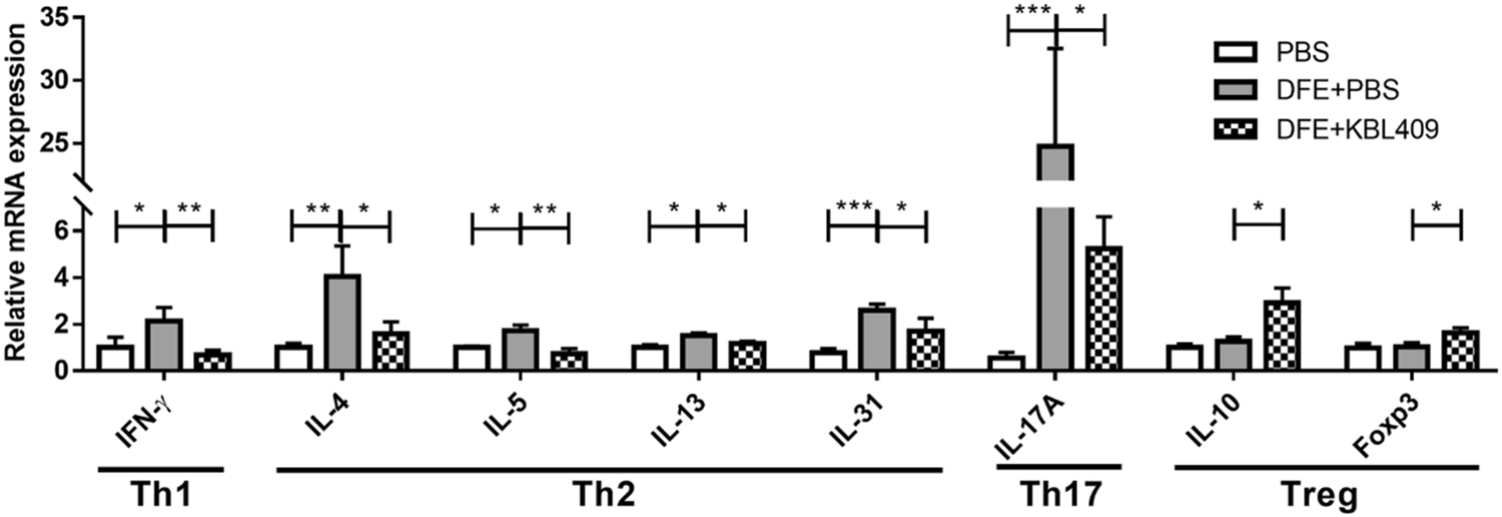
Effects of *L. acidophilus KBL409* on mRNA levels of cytokines and Foxp3 in AD-induced mice. Data are suggested as the mean ± SD of three independent experiments. Asterisks indicate a statistically significance (*P < 0.05; **P < 0.01; ***P < 0.001; the Mann–Whitney *U* test compared to the DFE + PBS-treated group)

Figure 2 showed effects of *L. acidophilus* KBL409 on mRNA levels of various cytokines and Foxp3. Compared to the DFE + PBS treated group, mice with *L. acidophilus* KBL409 administration had a significantly lower mRNA level of Th1 cytokine IFN-γ (0.68 ± 0.54 fold change; *P* < 0.01). Moreover, mRNA levels of Th2 and Th17 cytokines, including IL-4, IL-5, IL-13, IL-31, and IL-17A, were significantly down-regulated in mice with *L. acidophilus* KBL409 compared to the DFE + PBS treated group. An increase in the anti-inflammatory cytokine IL-10 (2.91 ± 1.92 fold change; *P* < 0.05) and an up-regulation of Foxp3 (1.62 ± 0.63 fold change; *P* < 0.05) were confirmed in AD-induced mice with *L. acidophilus* KBL409 administration.

### Effects of* L. acidophilus* KBL409 on Cecal Microbiota

**Fig. 3:**
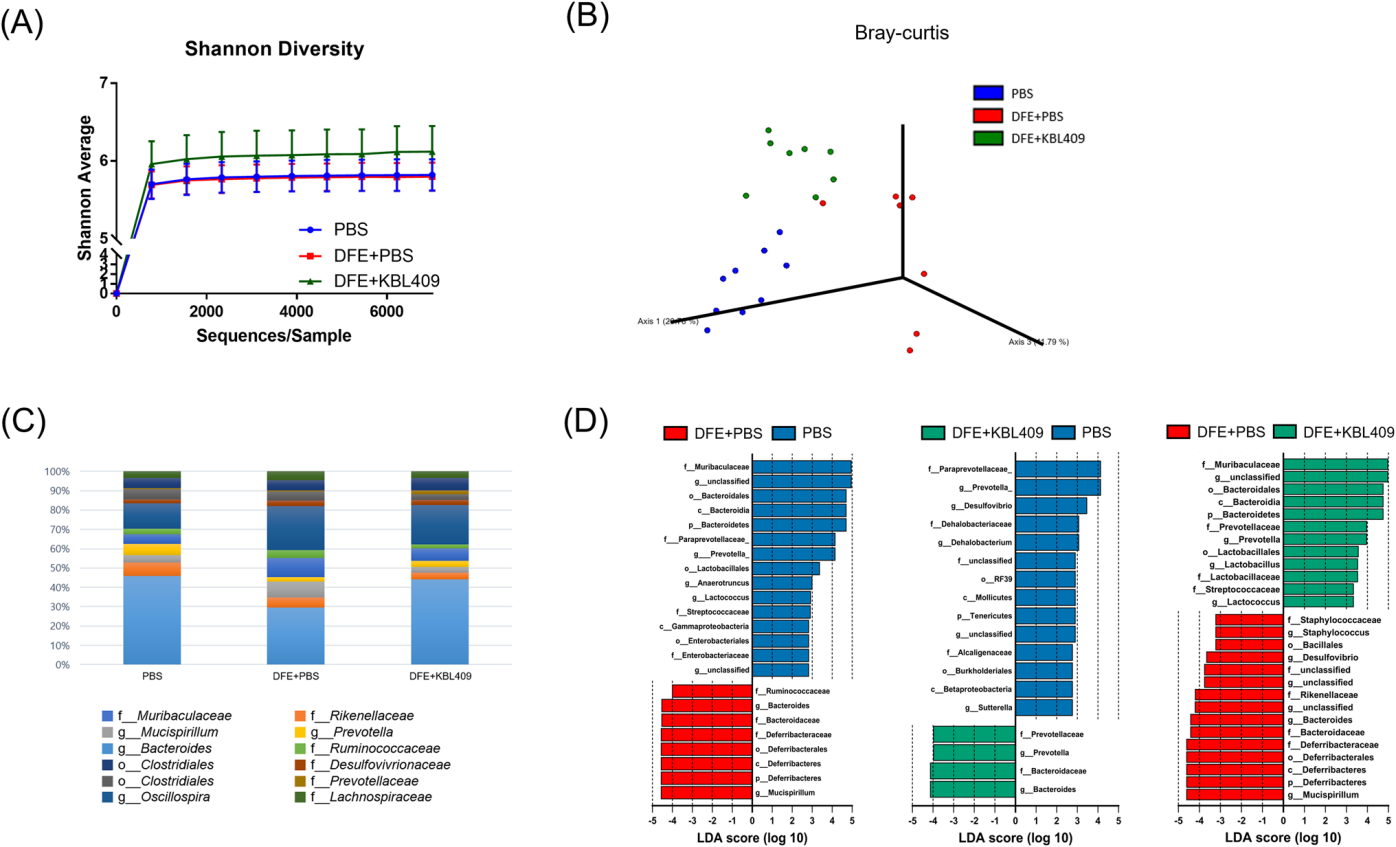
Effects of *L. acidophilus* KBL409 on cecal microbiota in AD-induced mice. **A** Shannon diversity indices; **B** Plots of the Bray–Curtis dissimilarity distance-based principal coordinates analyses; **C** Taxonomic structures of cecal microbiome in experimental groups; **D** Comparisons of significantly different taxa in experimental groups determined by LEfSe analyses (threshold > 2.5)

Figure 3 summarizes effects of *L. acidophilus* KBL409 administration on cecal microbiota of AD-induced mice. DFE + *L. acidophilus* KBL409 mice had higher bacterial diversities and discovered different bacterial communities compared to the DFE + PBS treated group (Fig. 3A, B). Mice with *L. acidophilus* KBL409 showed higher relative abundances of family *Muribaculaceae* and genera *Prevotella* and *Lactobacillus* than in the DFE + PBS treated group (Fig. 3C). However, in the DFE + PBS treated group, genera *Bacteroides*, *Mucispirillum,* and *Staphylococcus* were abundant species in cecum compared to other groups (Fig. 3D).

### Effects of *L. acidophilus* KBL409 on Predicted Metabolic Pathways and Various Metabolites in AD-Induced Mice

**Fig. 4:**
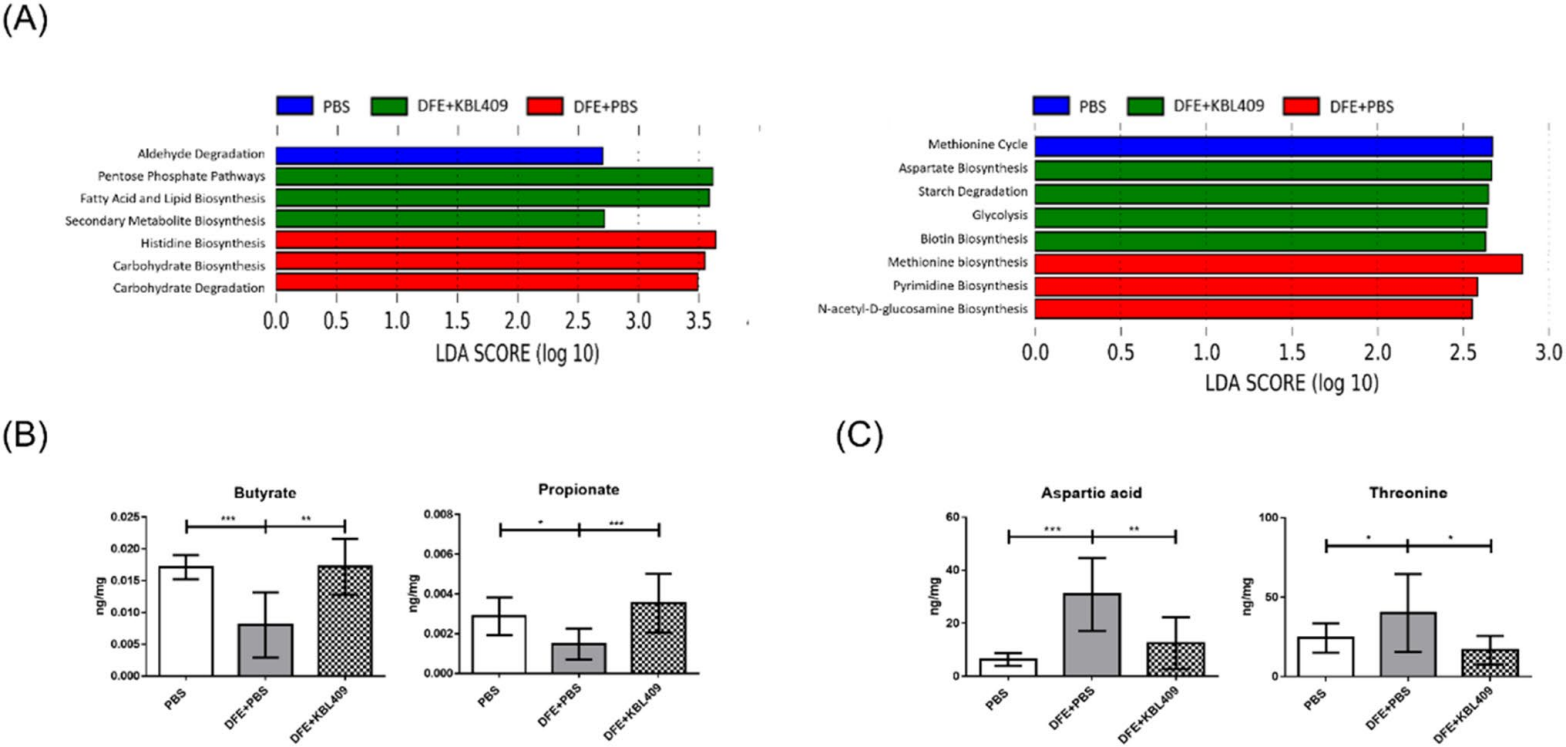
Effects of *L. acidophilus* KBL409 on predicted metabolic pathways and metabolite concentrations in cecum of AD-induced mice. **A** Predicted metabolic pathways determined by PICRUSt2 analyses; **B** Butyrate and propionate; **C** Aspartic acid and threonine. When appropriate, data are suggested as the mean ± SD of three independent experiments. Asterisks indicate a statistically significance (*P < 0.05; **P < 0.01; ***P < 0.001; the Mann–Whitney *U* test compared to the DFE + PBS-treated group)

In DFE + PBS treated mice, activities of predicted metabolic pathways for histidine, pyrimidine, and carbohydrate biosynthesis, as well as carbohydrate degradation, were elevated (Fig. 4A). Besides, increases in biosynthesis pathways for AAs, fatty acids, lipids, and secondary metabolites were discovered in mice with *L. acidophilus* KBL409 (Fig. 4A).

Concentrations of butyrate (0.018 ± 0.0044 ng/mg; *P* < 0.01) and propionate (0.0035 ± 0.0015 ng/mg; *P* < 0.001) were significantly higher in the DFE + *L. acidophilus* KBL409 treated group than DFE + PBS treated mice (Fig. 4B). DFE + *L. acidophilus* KBL409-treated mice had significantly lower concentrations of aspartic acid (12.50 ± 9.77 ng/mg; *P* < 0.01) and threonine (16.60 ± 9.08 ng/mg; *P* < 0.05) compared to the DFE + PBS treated group (Fig. 4C).

## Discussion

In this study, we found that oral administration of *L. acidophilus* KBL409 for 28 days ameliorated development of various clinical symptoms of AD, including erythema/hemorrhage, scaling/dryness, edema, and excoriation/erosion, and reduced IgE concentration (Fig. 1). Repeated topical applications of DFE exacerbate histological changes in the skin, such as increased epidermal thickness and up-regulation of IgE, and trigger immune cell infiltration into skin lesions (Lee et al., 2016; Matsuoka et al., 2003). Upon stimulation of allergens, dendritic cells (DCs) induce the differentiation of naïve T cells into Th2 cells and exacerbate skin inflammation by binding to IgE through its high-affinity receptor FcεRI (Peng & Novak, 2015). Skin infiltration of Th2 cells activates sensory nerves and induces B cells to produce IgE, mast cells, and eosinophils, which can release various inflammatory cytokines and chemokines (Fania et al., 2022; Lee et al., 2016; Poulsen & Hummelshoj, 2007). Based on the above mechanisms, our results suggest that *L. acidophilus* KBL409 improves AD symptoms by reducing both the IgE concentration and immune cell infiltration in the skin.

DFE + PBS treated mice showed significant increases in various mRNA levels of Th1, Th2, and Th17 cytokines in skin tissues, indicating chronic-phase AD (Fig. 2) (Fania et al., 2022; Peng & Novak, 2015). These cytokines activate of various immune cells, potentially leading to severe skin inflammation (He & Guttman-Yassky, 2019). Especially, IL-31, which is mainly released from activated Th2 cells, induces pruritus and lichenification during the development of AD (Szegedi et al., 2012). In the present study, *L. acidophilus* KBL409 administration induced high levels of Foxp3 and IL‐10 in skin of AD-induced mice (Fig. 2). Regulatory T cells (Tregs) control abnormal immune responses to inhibit interactions between DCs and effector T cells and activation of allergen-specific Th2 cells (Palomares et al., 2010). A Treg-associated transcription factor Foxp3 and an anti-inflammatory cytokine IL‐10 exert immunomodulatory effects by inhibiting T-cell proliferation (Poulsen & Hummelshoj, 2007; Vieira et al., 2004). Therefore, *L. acidophilus* KBL409-induced modulations in various cytokines and a Treg-associated transcription factor could be important effects to ameliorate AD.

*Lactobacillus acidophilus* KBL409 administration altered the gut microbiota and predicted metabolic pathways in AD-induced mice (Fig. 3). Bacterial diversities in DFE + *L. acidophilus* KBL409 treated mice were clearly increased compared to DFE + PBS treated mice and distinctively clustered than the other groups (Fig. 3A and 3B). Previous study has been reported that patients with AD had lower bacterial diversities than healthy individuals (Ye et al., 2021). Moreover, previous studies have been reported that the relative abundance of family *Muribaculaceae* and genus *Prevotella*, which were increased in DFE + *L. acidophilus* KBL409 treated mice, were positively correlated with the alleviation of AD-like symptoms in mice (Kim et al., 2019a, 2020, 2022a). Genus *Prevotella* can promote beneficial health effects, including improvement of glucose metabolism and propionate production (Precup & Vodnar, 2019). In contrast, same as the results of previous studies, abundances of genera *Bacteroides*, *Mucisiprillum*, and *Staphylococcus* were high in the DFE + PBS treated group (Fig. 3C and 3D) (Kim et al., 2019a, 2020; Watanabe et al., 2003). High abundances of genus *Bacteroides* have been discovered in patients with AD (Lucke et al., 2006) and abundances of genus *Mucisiprillum* were positively correlated with various in vivo colitis models (Loy et al., 2017). Previous study also has been suggested that high occurrences of genus *Staphylococcus* were discovered in intestine and skin of patients with AD (Watanabe et al., 2003). Taken together, our findings suggest that *L. acidophilus* KBL409 administration could ameliorates AD by increasing gut microbiota diversity and altering gut microbiota composition.

Several biosynthesis pathways, including AAs, fatty acids, lipids, and secondary metabolites, were positively correlated with *L. acidophilus* KBL409 administration (Fig. 4A). Moreover, high concentrations of propionate and butyrate were discovered in DFE + *L. acidophilus* KBL409 treated mice (Fig. 4B). SCFAs (e.g., acetate, propionate, and butyrate) are gut microbiota-produced metabolites that facilitate adenosine triphosphate (ATP) production, maintain the intestinal barrier, and regulate immune response (He et al., 2020; Portincasa et al., 2022). SCFAs have anti-inflammatory effects by promoting Treg differentiation (Vieira et al., 2004). AAs, which are mainly produced via gut microbiota, have major roles for host physiology and immunity and serve as substrates for SCFA synthesis (Dai et al., 2011; Neis et al., 2015; Rooks & Garrett, 2016). Our results also suggested that *L. acidophilus* KBL409 administration can affect production of threonine, which is the major substate of butyrate and involves propionate synthesis (Fig. 4C). However, further longitudinal studies with correlations between *L. acidophilus* KBL409 administration and dietary fiber fermentation via gut microbiota, which is the major source of SCFAs in gut (Canani et al., 2011; McNabney & Henagan, 2017), should be performed to elucidate mechanisms of *L. acidophilus* KBL409 to modulate immune responses using SCFAs fully.

In conclusion, oral administration of *L. acidophilus* KBL409 significantly ameliorated the progression of AD in a mouse model by modulating the immune responses and restoring the gut microbiota. Therefore, our results suggest that *L. acidophilus* KBL409 has potential for AD treatment and further studies with longitudinal human challenges using high concentrations of *L. acidophilus* KBL409 or consortia using other probiotic strains are needed to establish its applications as the major component of functional foods and/or therapies.

### Supplementary Information

Below is the link to the electronic supplementary material.

Supplementary file1 (PDF 228 KB)

## Data Availability

The data supporting the findings in this study are available from the corresponding author upon reasonable request.
