# Supplementary material for: Lactobacillus acidophilus KBL409 Ameliorates Atopic Dermatitis in a Mouse Model
Source: J Microbiol. 2024 Feb 22;62(2):91–9. doi: 10.1007/s12275-024-00104-5 (PMC11021314; doi:10.1007/s12275-024-00104-5)
Supplement: Supplementary file 1 — Supplementary file1 (PDF 228 KB) [file 12275_2024_104_MOESM1_ESM.pdf]

Table S1. Calculated dermatitis score (Maximun score = 12)

Content	Score	Clinical severity
**Erythema/Hemorrhage**	0	None
	1	Mild
	2	Moderate
	3	Severe
**Scaling/Dryness**	0	None
	1	Mild
	2	Moderate
	3	Severe
**Edema**	0	None
	1	Mild
	2	Moderate
	3	Severe
**Excoriation/Erosion**	0	None
	1	Mild
	2	Moderate
	3	Severe

Table S2. Primers used in this study

Target	Sequence	Reference
**HPRT**	Fw: 5′-TTA TGG ACA GGA CTG AAA GAC-3′	
	Rv: 5′-GCT TTA ATG TAA TCC AGC AGG T-3′	
**Foxp3**	Fw: 5’-CCC ATC CCC AGG AGT CTT G-3’	
	Rv: 5’-CCA TGA CTA GGG GCA CTG TA-3’	
**IFN-γ**	Fw: 5′-TCA AGT GGC ATA GAT GTG GAA GAA-3′	
	Rv: 5′-TGG CTC TGC AGG ATT TTC ATG-3′	
**IL-4**	Fw: 5’-ACA GGA GAA GGG ACG CCA-3’	Kwon et al. (2010)
	Rv: 5’-GAA GCC CTA CAG ACG AGC TCA-3’	
**IL-5**	Fw: 5′-TCC AAT GCA TAG CTG GTG ATT T-3′	
	Rv: 5′-AGC ACA GTG GTG AAA GAG AC-3′	
**IL-10**	Fw: 5′-TCA TTT CCG ATA AGG CTT GG-3′	
	Rv: 5′-ATA ACT GCA CCC ACT TCC CA-3′	
**IL-13**	Fw: 5’-GCA ACA TCA CAC AGG ACC AGA-3’	
	Rv: 5’-GTC AGG GAA TCC AGG GCT AC-3’	
**IL-17A**	Fw: 5′-TTC ATC TGT GTC TCT GAT GCT-3′	
	Rv: 5′-TTG ACC TTC ACA TTC TGG AG-3′	
**IL-31**	Fw: 5′-ATA CAG CTG CCG TGT TTC AG -3′	Takaoka et al. (2006)
	Rv: 5′- AGC CAT CTT ATC ACC CAA GAA -3′	

[a]Fw represents sequences of a forward primer

[b]Rv represents sequences of a reverse primer

References

Kwon, H. K., Lee, C. G., So, J. S., Chae, C. S., Hwang, J. S., Sahoo, A., Nam, J. H., Rhee, J. H., Hwang, K. C., & Im, S. H. (2010). Generation of regulatory dendritic cells and $CD4^+Foxp3^+$ T cells by probiotics administration suppresses immune disorders. *Proceedings of the National Academy of Sciences of the USA*, *107*, 2159–2164.

Takaoka, A., Arai, I., Sugimoto, M., Honma, Y., Futaki, N., Nakamura, A., & Nakaike, S. (2006). Involvement of IL-31 on scratching behavior in NC/Nga mice with atopic-like dermatitis. *Experimental Dermatology*, *15*, 161–167.
